# Terrestrial Water Storage in African Hydrological Regimes Derived from GRACE Mission Data: Intercomparison of Spherical Harmonics, Mass Concentration, and Scalar Slepian Methods

**DOI:** 10.3390/s17030566

**Published:** 2017-03-10

**Authors:** Ashraf Rateb, Chung-Yen Kuo, Moslem Imani, Kuo-Hsin Tseng, Wen-Hau Lan, Kuo-En Ching, Tzu-Pang Tseng

**Affiliations:** 1 Department of Geomatics, National Cheng Kung University, No. 1, University Road, Tainan City 701, Taiwan; kuo70@mail.ncku.edu.tw (C.-Y.K.); moslem.imani62@gmail.com (M.I.); p68001021@mail.ncku.edu.tw (W.-H.L.); jingkuen@mail.ncku.edu.tw (K.-E.C.); 2 National Authority for Remote Sensing and Space Sciences, Cairo, 1564 Alf Maskan, Egypt; 3 Center for Space and Remote Sensing Research, National Central University, Taoyuan 32001, Taiwan; khtseng@csrsr.ncu.edu.tw; 4 Cooperative Research Centre for Spatial Information, Australia, Level 5, 204 Lygon St, Carlton VIC 3053, Australia; tzupang.tseng@gmail.com; 5 Geoscience Australia, Australia, Cnr Jerrabomberra Ave and Hindmarsh Drive, Symonston ACT 2609, Australia

**Keywords:** terrestrial water storage, GRACE, spherical harmonics, global mascon, Slepian basis, Africa basins

## Abstract

Spherical harmonics (SH) and mascon solutions are the two most common types of solutions for Gravity Recovery and Climate Experiment (GRACE) mass flux observations. However, SH signals are degraded by measurement and leakage errors. Mascon solutions (the Jet Propulsion Laboratory (JPL) release, herein) exhibit weakened signals at submascon resolutions. Both solutions require a scale factor examined by the CLM4.0 model to obtain the actual water storage signal. The Slepian localization method can avoid the SH leakage errors when applied to the basin scale. In this study, we estimate SH errors and scale factors for African hydrological regimes. Then, terrestrial water storage (TWS) in Africa is determined based on Slepian localization and compared with JPL-mascon and SH solutions. The three TWS estimates show good agreement for the TWS of large-sized and humid regimes but present discrepancies for the TWS of medium and small-sized regimes. Slepian localization is an effective method for deriving the TWS of arid zones. The TWS behavior in African regimes and its spatiotemporal variations are then examined. The negative TWS trends in the lower Nile and Sahara at −1.08 and −6.92 Gt/year, respectively, are higher than those previously reported.

## 1. Introduction

Obtaining reliable terrestrial water storage (TWS) estimates from the Gravity Recovery and Climate Experiment (GRACE) is challenging but crucial for studying hydrological cycles and climate change, especially in regions with limited freshwater resources, such as Africa. GRACE was launched in March 2002 into a near-polar orbit at an elevation of 450 km to measure the temporal Earth gravity field caused by geophysical phenomena [1,2]. GRACE has many applications in quantifying different parameters of the hydrological cycle, including quantifying TWS variations [3,4,5,6], groundwater depletion [7,8,9,10], evapotranspiration [11,12], TWS extremes [13,14,15], freshwater discharge [16,17], climate and human interactions [10,18], and TWS-driven deformation [19,20,21]. Furthermore, the mission has been used to calibrate and evaluate land hydrological models [22,23].

Over the last decade, the mass flux from this mission has been represented by monthly global solutions based on spherical harmonics (SH) [24], and by mascon solutions (the Jet Propulsion Laboratory (JPL) release, herein) [25,26]. The traditional SH solutions suffered from measurement errors represented by non-physical longitudinal “stripes” and obtained as the result of the north-south ground track orientation. These stripes obscure the east-west gravity gradient and are associated with high-degree SH—the most valuable coefficients for studying geophysical phenomena [27]. In addition, given the satellite altitude at 450 km, GRACE is sensitive to mass variations over areas of ≥250,000 km^2^; however, its resolution is dubious for regions smaller than this threshold [28]. These errors and limitations have been reduced by applying different spectral filters and smoothing approaches as post-processing treatments. The most widely applied strategy is to implement the de-stripe algorithm [29] and classic Gaussian smoothing [30]. Filter selection is critical because of the signal characteristics, location, and shape of the region of interest (ROI) [31]. In ROI studies, solutions are restricted within the ROI by building concentrated basis function (CBF) [29,32]. However, these approaches have drawbacks. For example, the filters may simultaneously reduce real north-south geophysical signals and increase the solution uncertainty [2,33]. The smoothing or spatial averaging increases biases inside the ROI and external leakage from the surrounding regions [33], and the CBF distorts the actual ROI shape in computation [28]. A common method used to restore the actual signal is to adopt scale factors that are based on a priori information from land surface models (LSMs) and can be applied to the filtered GRACE solutions at grid or ROI scales [29,34]. Gridded GRACE-TWS derived from SH solutions are obtained by implementing this strategy with the Community Land Model 4.0 (NCAR-CLM4.0) at a 1° × 1° grid scale [34]. In mascon solutions, the measurement errors are minimized by designing Bayesian constraints as the gravity state variable matrix for known geophysical information during the range rate inversion [25]. The JPL-mascon solution is expressed as a collection of spherical caps smoothed at a resolution of 3° × 3°. The smoothing weakens the submascon objects and still requires a separate approach to strengthening the weakened sub-mascon signal [25] by implementing the same scale factor of the CLM4.0 model at a 0.5° × 0.5° grid scale.

The scalar Slepian localization method is recently suggested as a post-processing approach for ROI studies. The method localizes the SH solutions efficiently onto new bandlimited functions (e.g., CBF). The new functions possess advantages over SH by being orthogonal over the globe and ROI, being less dependent on filtering, and smoothing and offering exerting control over the SH range [35,36]. As a result, the leakage errors are attenuated from the surrounding areas, and spatial resolutions inside the ROI are increased [35]. The method has been applied to different geophysical and planetary phenomena, such as: (1) mapping the spatiotemporal loss of the ice shields [35]; (2) earthquake-derived gravity changes [37,38]; (3) deriving the TWS in the Amazon basin [39] and high plain aquifer [28]; (4) estimating the ocean bottom pressure signal [39]; and (5) obtaining a high local resolution model for the Mars south pole’s magnetic field [40]. Among the different CBF methods, the scalar Slepian localization approach can deduce a reliable TWS estimate for small and irregularly shaped ROI [28].

The performance of the Slepian CBF in comparison with JPL-mascon and SH solutions at regional or ROI scale has not been investigated yet. In the current research, we achieve three main objectives: first, we analyze GRACE errors and scale factors that are applied in SH and JPL-mascon solutions for African hydrological regimes. Second, TWS anomalies are determined by implementing the Slepian method, and the result is compared with the SH and JPL-mascon based estimates. Third, the TWS changes in African regimes are carefully assessed. The present assessment is significant for the sustainable management of small water resources in a region where approximately 35% of the population (85% in the rural areas) has difficulty accessing freshwater [41,42].

## 2. Materials and Methods

### 2.1. Study Area

**Figure 1:**
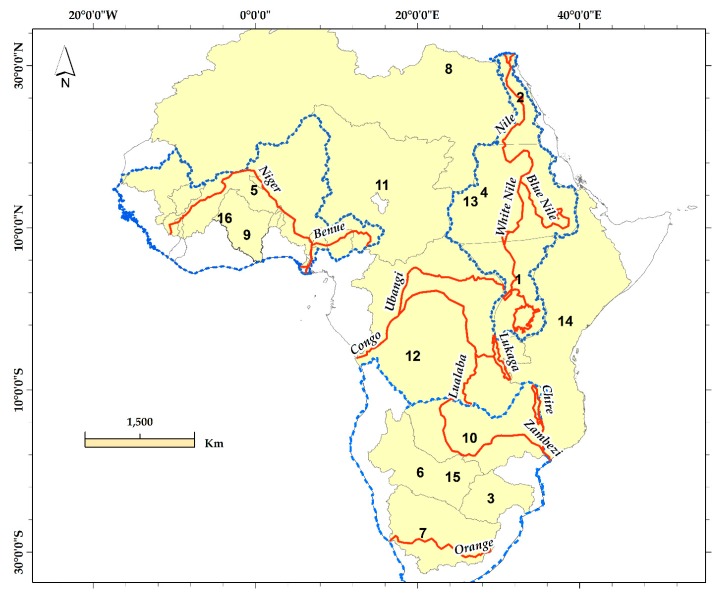
Major hydrological regimes, including drainage basins and arid and semi-arid zones: (1, Higher Nile; 2, Lower Nile; 3, Limpopo; 4, Middle Nile; 5, Niger; 6, Okavango; 7, Orange; 8, Sahara; 9, Volta; 10, Zambezi; 11, Chad; 12, Congo; 13, Nile; 14, Rift Valley; 15, South Africa; 16, West Africa). Blue dashed lines outline the mega regimes (Nile, West Africa, and South Africa), and the river systems are marked in red lines.

We study 16 regions that represent major African hydrological regimes (i.e., ROIs) of different sizes and hydrological conditions (topography and climate) (Figure 1). The ROI shapes are derived from the hydroshed of Shuttle Radar Topography Mission elevation data with a resolution of three-arc seconds (approximately 90 m) at the equator [43]. According to the global Aridity Index (AI), Africa experiences different climate domains that vary from hyper-arid to humid (Table 1). 

The AI identifies the aridity of the region using the ratio of the mean annual precipitation to the mean annual evapotranspiration [44]. Hyper-arid, arid, semi-arid, dry sub-humid, and humid domains have ranges of <0.03, 0.03–0.2, 0.2–0.5, 0.5–0.65, and >0.65, respectively [45]. The mega regimes (Nile, West Africa, and South Africa) experience different hydrological conditions. Inferring the TWS changes in the entire regime in a single time series misrepresents the behavior of small basins in the regime. Therefore, we model small regimes independently and included the entire mega regime in different scenarios.

### 2.2. Theory and Practice

#### 2.2.1. GRACE-Estimated Errors and Scale Factors

SH GRACE errors include measurement “stripes” and leakage errors. The measurement errors (*σ_m_*) are shown as non-physical longitudinal strips that originate during range rate inversion. According to Wahr et al. [27], the errors can be estimated in two steps: first, the SH uncertainties are derived from the calibrated errors which are provided with the GRACE data release. These errors are fitted to remove the constant and annual component and then scaled with a degree-dependent factor to ensure good agreement with the smoothed calibrated errors. Second, the residuals are used to estimate the mass errors, with the Gaussian averaging at a smoothing radius of 750 km for the global scale (Equation (4) in Wahr et al. [27]). The errors are smaller near the high latitudes than near the low latitudes, with the global average of approximately 21 mm. They vary by month, are normally distributed, and depend on the smoothing radius and size of the region [27]. Leakage errors (σ*_l_*) represent the deviation of filtered and unfiltered signals. They can be quantified by the RMS difference between the filtered and unfiltered mean of the CLM4.0 model solutions [34]. These errors are highly correlated at the basin scale and cannot be simply obtained by averaging the gridded points over the basin. We follow the method described by Landerer and Swenson [34], in which the error variance is calculated based on the error covariance between neighboring points ($x_{i},x_{j}$) and fixed Gaussian window ($w_{i}w_{j}$). The window is specified by the distance that has the half of the maximum function value:

(1) $$\mathrm{var}=\overset{N}{\underset{i=1}{\sum }}\overset{N}{\underset{j=1}{\sum }}w_{i}w_{j}Cov\;\left(x_{i},x_{j}\right)$$

The total errors are obtained by summations of *σ_m_* and *σ_l_* in quadrature. The posterior scale factor from the free NCAR-CLM4.0 model is applied to restore the actual signal. The TWS from the CLM4.0 model is converted to SH. Then, the SH is filtered with the same filter applied to the GRACE SH solutions and remapped into the SH and mascon resolutions. The scale factor (k) is obtained by least square regression through fitting the difference between the filtered ($∆S_{T}$) and unfiltered ($∆S_{F}$) signals [34]. The objective function M must be minimized:

(2) $$M=\sum^{\text{​}}{\left(∆S_{T}-k∆S_{F}\right)}^{2}$$

The GRACE errors and the scale factor are provided independently by the GRACE Tellus website [46]. Table 1 shows the SH solution errors, and the scale factor is obtained for the ROIs under investigation.

#### 2.2.2. Scalar Slepian Localization

The Slepian localization approach relies on transforming the traditional SH basis into a localized orthogonal basis [47]. The idea is to find a group of functions $\hat{h}$ that maximize the potential energy over the ROI and become zero outside. This step can be conducted through solving the eigenvalues problem of the SH over the ROI $D\hat{h}=\lambda \hat{h}$, where D is the matrix of the Legendre polynomial products and λ is the eigenvalue numbers. The number of the new orthogonal functions N depends only on the maximum SH degree $L_{max}$ and area A of the ROI, $N={\left(L_{max}+1\right)}^{2}\frac{A}{4\pi }$. The method fully controls the SH range to limit the noise contaminations of the SH and achieve a balance between the spatial and spectral resolutions [48]. For the detailed mathematical formulation of the method, statistical performance and comparison with SH and computational efficiency are described by Simons [49]. We use the bandwidth of SH solutions from the Center for the Space Research (CSR) solutions of $L_{max}=$ 60, and coordinates of every ROI to obtain a new orthogonal basis up to 3721. The new orthogonal functions range between 1 and −1 based on the value of λ (that is, the efficiency of the CBF) to represent the GRACE data inside and outside the ROI. The function has a value near unity: the more favorable to restrict the GRACE solutions to the ROI and vice versa. We determine the CBFs of λ≥0.6, where the signal is maximized only inside the ROI and becomes zero outside. The number of efficient CBFs varies with the size and shape of the ROI under study (Table 2).

### 2.3. GRACE Data and Processing

The GRACE-gridded TWS is available at a resolution of 1° × 1° and is derived from the RL05 of SH with the bandwidth of 60 from the CSR, GeoForschungsZentrum, and JPL data centers. The solutions are obtained after being processed with the following steps [1]. (1) Atmosphere and ocean signals are removed by the ECMWF IFS and AOD1B background model; (2) The C_20_ by GRACE is replaced with the C_20_ derived from satellite laser ranging because of the large uncertainty in C_20_ by GRACE [50]. The degree one coefficients estimated by Swenson et al. [51] are added; (3) Glacial isostatic adjustment is corrected by Geruo et al.’s [52] model; (4) The correlated errors are minimized with the destriping filter; (5) The Gaussian filter is implemented with a radius of 300 km to reduce highly correlated errors. In the current study, we obtain 149 monthly solutions from the three centers covering the period between April 2002 and December 2015. The solutions are averaged and scaled with the scale factor of the NCAR-CLM4.0 model with the SH-TWS as the final product. For JPL-mascon solutions, the data are processed similar to SH-TWS, except step 4 because of applying the prior Bayesian constraints of the spherical caps. CLM4.0 derived scale factors are also adopted at a resolution of 0.5° × 0.5° grid points with the MSC-TWS the result. In the case of the Slepian method, we use the CSR SH of up to 60 and process for the same first three steps of the SH-TWS. Then, the potential solutions are transformed into TWS [2]. Finally, the TWS solutions are projected to the effective CBFs in every ROI.

### 2.4. Analysis and Model

We apply the Karhunen-Loève transform (KLT) to derive the dominant spatiotemporal modes of TWS for SH-TWS and MSC-TWS. The details of the KLT can be found in Jolliffe [53]. We organize the data of SH-TWS and MSC-TWS in ***D*** matrices with a size of *m × n*, where *m* is the number of the variables at 1° or 0.5° interval and *n* is the number of observations (149 months). The ***D*** matrices are decomposed by the singular value decomposition algorithm to obtain empirical orthogonal function (EOF in Table 2) modes and principal components. The number of extracted modes range between 2 and 4 and vary by region size (Table 2). The first mode explains approximately 70%–80% of the variability in the TWS of large regions. The second mode explains approximately 10%–20%. The first mode accounts for 85%–92% of the TWS variability in small areas while the second explains 5%–15%. We reconstruct 98% of the variability in data by combining the approximated data of the high ranks, which are indicated by the maximum eigenvalues.

**Figure 2:**
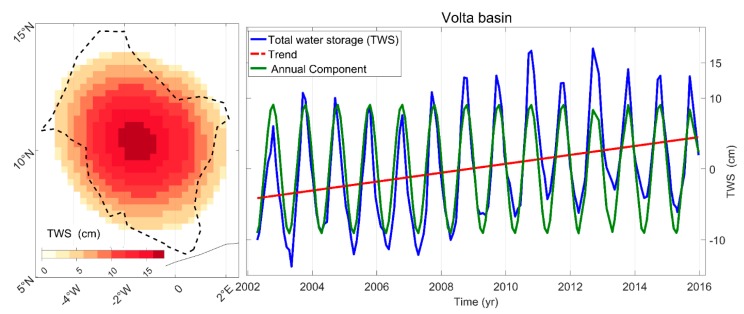
(**Left**) spatial patterns of the equivalent water height in the Volta basin derived from the best concentrated Slepian CBF; (**Right**) time series of the TWS between April 2002 to December 2015 on the blue line. The trend and annual component of the TWS are marked in dashed red and green lines, respectively.

For the Slepian-based estimates, the TWS is projected into the CBFs in every ROI to yield the Slepian coefficients as one coefficient per month in every CBF. Then, the coefficients are integrated over the region to obtain the best estimate of the TWS. These summations are used as SL-TWS for comparison with SH-TWS and MSC-TWS. Figure 2 shows an example of the anomalies in the TWS of the Volta basin. 

These anomalies are obtained through the Slepian localization method. The long-term trend ($\beta_{1}$), annual component ($\beta_{2}$, $\beta_{3}$), and semi-annual component ($\beta_{4}$, $\beta_{5}$) can be estimated by fitting the TWS anomalies (*Y*) to Equation (3) with the least squares regression:

(3) $$Y\left(t\right)=\beta_{0}+\beta_{1}t+\beta_{2}cos\left(2\pi t\right)+\beta_{3}sin\left(2\pi t\right)+\beta_{4}cos\left(4\pi t\right)+\beta_{5}sin\left(4\pi t\right)+\varepsilon ,$$

where $\beta_{0}$ is the bias term, and ε is the random error. The estimated parameters are shown in Table 3.

To evaluate the performance of the three TWS estimates, we calculate the Pearson correlation coefficient (R) and Nash-Sutcliffe efficiency (NSE) [54]. NSE can be used to assess the model accuracy and ranges from −∞ to 1. NSE = 1 implies a perfect correspondence between the compared models, whereas NSE = 0 signifies that the second model is as accurate as the mean of the first. NSE < 0 indicates that the first model is a better predictor than the second. The correlation coefficient is sensitive to the phase but not to the amplitude of the time series, whereas NSE is sensitive to the phase, amplitude, and means of the compared time series.

Given the scarcity of in situ ground data in Africa, validating GRACE measurements is challenging. Herein, the only available ground station of the water level observations is in the Okavango basin [55], which is located at the Rundu beach (19.76° E, −17.90° N). The data from this station cover the period between 2002 and 2011. The correlation coefficients between the ground data and the three estimates from GRACE of the TWS are calculated.

## 3. Results

### 3.1. GRACE Errors and CLM4.0 Scale Factor in African Regimes

**Figure 3:**
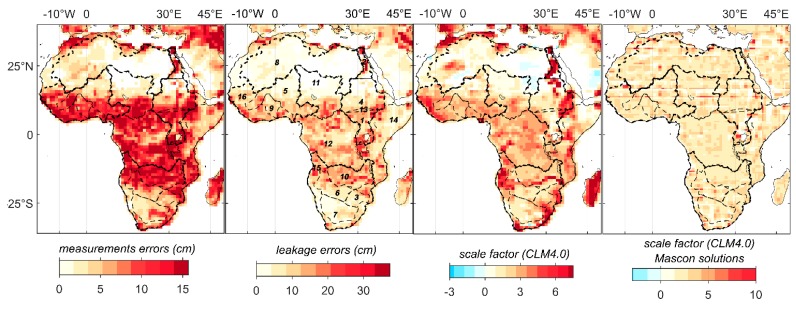
Spatial patterns of GRACE errors and CLM4.0 scale factors in Africa.

Figure 3 shows the spatial patterns of the measurement errors, leakage errors, and CLM4.0 scale factors (*k*_SH_, *k*_MS_) in Africa. The errors at the ROI scale are presented in Table 1 (columns 5–7). Figure 3 and Table 1 clearly show that the leakage errors are smaller than the measurement errors and represent 41%–43% of the total errors. Comparing ROIs of similar size and different climate domains reveals that the small-sized arid ROI presents larger errors than the small dry sub-humid ROI. For example, the lower Nile (arid ROI) experiences a high error level of (*σ_l_* = 13.25 mm, *σ_m_* = 25.43 mm) than Volta (dry semi-humid ROI) with (*σ_l_* = 5.81 mm, *σ_m_* = 13.61 mm). However, the scale factor obtained from the CLM4.0 is smaller for the lower Nile (k_SH_ = 0.38, and k_MS_ = 0.88) than that for Volta (k_SH_ = 1.36, k_MS_ = 1.34). Similar results are observed for large-sized ROIs, such as the arid Nile basin, with large errors (*σ_l_* = 6.25 mm, *σ_m_* = 13.44 mm) and low scale factors (k_SH_ = 1.15, k_MS_ = 0.97) compared with the humid Congo basin that has small errors (*σ_l_* = 5.38, *σ_m_* = 10.83 mm) and high scale factors (k_SH_ = 1.24 and k_MS_ = 1.09). The effects of the leakage on the TWS annual components of the ROI’s TWS can also be observed in small-sized arid ROIs. For example, the Limpopo and Okavango ROIs (semiarid) experience phase shifts of up to 10° due to sharing boundary with the Zambezi (dry sub-humid) basin (Table 3). The scale factor obtained from CLM4.0 for the MSC-TWS is smaller than that obtained for the SH-TWS, as the mascon-based estimate does not experience the same error level of the SH-based estimate.

### 3.2. Statistical Performance of the TWS Estimates

Table 2 shows the statistical performance including the correlation coefficients (R) and NSE test obtained from an intercomparison of the three estimates. (NSE1, R1) refer to the comparison of SL-TWS versus SH-TWS, (NSE2, R2) for SL-TWS versus MSC-TWS, and (NSE3, R3) for SH-TWS versus MSC-TWS. The correlation coefficients indicate that the agreement between SL-TWS and SH-TWS is higher than that between MSC-TWS and SL-TWS or SH-TWS in all ROIs. Highly positive coefficients of 0.83–0.93 are observed for large-sized and dry sub-humid to humid ROIs in Nile, Congo, West Africa, and South Africa. Correlation coefficients of 0.62–0.89 are observed in medium-sized ROIs in either dry sub-humid or arid (e.g., Zambezi and Niger). For the small-sized ROIs, the agreement of SL-TWS and SH-TWS is higher for humid ROI than that for dry ROI and shows coefficients of 0.85 and 0.73 in the Volta and Okavango basins for R1.

The NSE test supports the correlation results, where the NSE1 coefficients are larger than those of NSE2 and NSE3. NSE1 efficiency ranges between 0.31 and 0.82 in all ROIs, indicating that the consistency between SL-TWS and SH-TWS estimates is better than that among both and MSC-TWS. The large-sized ROI of either humid or arid has the best matching between the three estimates, with an efficiency higher than 0.52 (e.g., Nile, South Africa, and West Africa). In medium and small-sized ROIs, the means of SL-TWS and MSC-TWS estimates are better predictors than that of SH-TWS and exhibit efficiency between 0.31 and 0. In small-sized arid ROI, the SL-TWS estimate is the best predictor, followed by the SH-TWS and MSC-TWS with an NSE > 0 (e.g., the lower Nile and Orange). Another comparison is made based on the fitting parameters (Table 3). The results show that MSC-TWS has the highest magnitude, followed by SH-TWS and SL-TWS. Validating with the water level of the ground station in the Okavango basin, the coefficients are 0.51, 0.43, and 0.26 for MSC-TWS, SL-TWS, and SH-TWS, respectively.

### 3.3. TWS Variations in Africa

**Figure 4:**
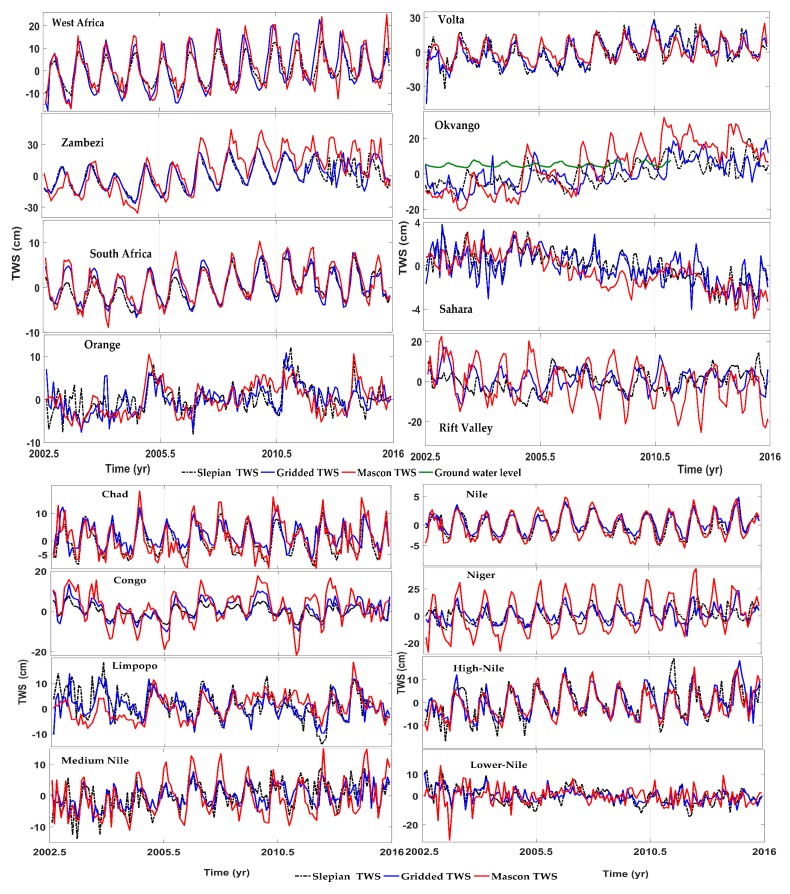
Time series for the three estimates in 16 African hydrological regimes.

**Figure 5:**
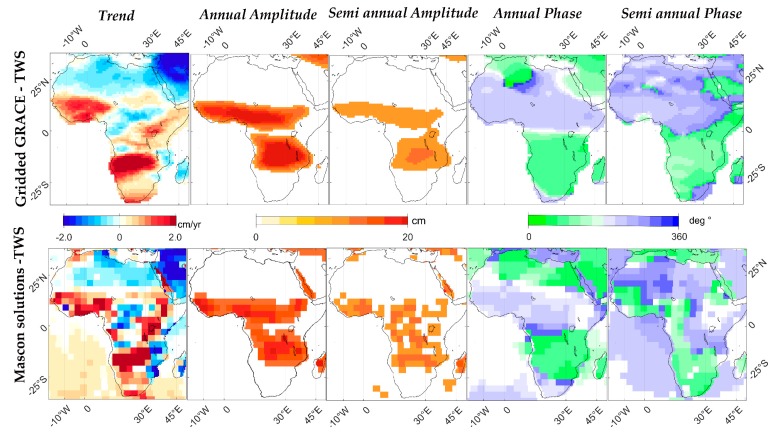
Spatial maps of TWS trend and periodic components of total water storage between April 2002 and December 2015 derived from SH and JPL-mascon solutions in Africa.

The fitting parameters of the three TWS estimates in African hydrological regimes for the long-term trend and periodic components are shown in Table 3. The time series are given in Figure 4, and the spatial maps are illustrated in Figure 5. Ongoing positive trends of TWS storage are observed in most of the ROIs, except for Sahara, Limpopo, and the lower Nile. The highest estimated trends are observed in West Africa (2.82 ± 0.9 cm/year for the Volta basin and 1.74 ± 0.4 cm/year for Niger), followed by equatorial East Africa (1.87 cm/year in the middle Nile) and South Africa (1.44 cm/year for the Okavango). The remaining ROIs present insignificant rates that are lower than 1 cm/year. Conversely, large negative trends are observed in the lower Nile with a rate of −2.36 cm/year, which equals to a volume of −1.08 Gt/year from the SL-TWS. Moreover, Sahara exhibits a depletion rate of nearly −1.02 ± 0.7 cm/year equivalent to the volume of −6.92 Gt/year. For the periodic components, the largest annual amplitude is observed in Volta at 14.30 cm, followed by Niger, Zambezi, and Congo at 9.35, 14.94, 6.8, and 5.64 cm, respectively. 

The higher Nile obtains the highest value for semi-annual amplitude at 5.51 cm. In arid zones such as Sahara and Orange, the annual amplitude shows a background noise that ranges from 0.14 cm to 2.32 cm.

## 4. Discussion

### 4.1. GRACE Error Assessment at African Hydrological Regimes

At the ROI scale, GRACE errors are functions of size, climate, and post-processing steps. According to the obtained results, the water content of the ROI may govern the error level more than the size of the ROI. Humid catchments present smaller errors compared with arid ROI, and large-sized dry ROIs exhibit smaller error level than small-sized arid ROIs. The reason is that the spatially varied TWS distribution in arid ROIs generates incoherent errors, unlike the humid ROIs where the TWS distribution is spatially coherent. The scale factors implemented from the CLM4.0 model and applied to arid ROIs are smaller than those for humid ROIs. The scale factor for a dry ROI is loosely estimated because the CLM4.0 model poorly represents the full components of TWS changes in arid ROIs (e.g., irrigation). Irrigation is the main use of freshwater in arid regions. The results agree well with similar TWS analyses and scale factors obtained from other LSM models, such as VIC, mosaic, and GLDAS [56]. However, these models simulate only the surface moisture and experience high uncertainty and spatiotemporal variations [34]. Therefore, relying only on the scale factor in deriving the actual SH-TWS signal particularly for arid ROIs is insufficient and can result in miscalculation when studying TWS extremes (floods or drought) or long-term groundwater depletion.

### 4.2. Comparison of SH-TWS, SL-TWS, and MSC-TWS

In comparing the three estimates, agreement and consistency at large humid ROIs are expected. Specifically, the size of the regime should be higher than the GRACE resolution threshold of approximately 450 km, and water content coherence should be high. The three estimates for large-sized ROI have similar values in trend and an annual amplitude component. Differences can be found in the ROI near the GRACE spatial limit, where Slepian and mascon estimates show similar larger magnitudes than SH. Large-sized ROIs also show a phase shift up to 5° between SH and Slepian estimations. The shift considerably decreases by approximately 3° for medium-sized dry sub-humid ROIs and increases differently in small-sized arid ROIs among the three estimates. This phase shift is ascribed to asymmetric leakage errors from the interaction among the regime’s surroundings, where high-aridity regimes gain signals from low-aridity regimes and vice versa. The small phase shift and relatively low reduction in amplitude of the SH-TWS estimate compared with those of the MSC-TWS and SL-TWS estimates attributed to the small smoothing radius of 300 km. However, increasing the smoothing filter radius to 800 km reduces the TWS annual amplitude between 25% and 40% and shifts the phase up to 10° [57]. In moderately sized ROIs, the phase shifting becomes substantial of up to 20°–30° and close to one month [58]. Therefore, the reliable TWS estimate based on the SH requires a balance between reductions in spatial resolutions and noises [28].

Leakage errors are highly reduced when the optimally localized function is used in Slepian estimations. The leaked signal is concentrated at the ROI boundary and becomes zero outside the ROI. With less dependence on smoothing and a priori hydrological model, the Slepian method becomes effective for monitoring TWS based on GRACE SH representations. This situation is especially true for arid zones, which experience high-level errors and poor LSM performance. In mascon solutions, leakage errors from the ocean to the land and vice versa are minimized by applying a coastline filter that reduces leakage errors by 50% from 2 cm to 1 cm in non-ice regions and by 80% in ice-covered regions from 10 cm to 2 cm [59]. For the leakages between hydrological basins, the combination of coastline filter and CLM4.0 scale factor have modestly reduced the leakage errors by 11%–30% in large-sized ROIs. For small-sized ROIs, the reduction becomes substantial by 38%–81% up to 9–19 mm [59]. Comparing the leakage error obtained from SH-TWS and simulations for Africa basins in Wiese et al. [59], mascon errors are smaller than SH errors by 30%–50% and the weakened signal has recovered by 7%–33% in large-sized ROIs and 35%–45% in small-sized ROIs [59].

### 4.3. TWS Recharge and Depletion in Africa

The negative trend of TWS storage in Sahara is −6.92 Gt/year and in the lower Nile is −1.08 Gt/year. These negative trends result from the overexploitation of groundwater for irrigation under the dry climate. Sahara has two transboundary fossil aquifers that represent shared freshwater stocks for the agricultural sectors of the neighboring countries. The northwest aquifer is partitioned among Tunisia, Libya, and Algeria, whereas the Nubian aquifer is shared among Egypt, Sudan, and Chad. The resolved depletion rate in the entire Sahara area is higher than that previously estimated for both aquifers. In the Nubian aquifer, the resolved rate is approximately −2.04 ± 0.99 × 10^9^ m^3^/year as reported by Sultan et al. [60] and is calculated to be −4.4 km^3^/year for the northwest aquifer [61]. Annual recharge is low from precipitation or between aquifers [62], indicating a small cumulative recharge rate of approximately 1.40 ± 0.90 m^3^/year in the northwestern aquifer. As a result of overexploitation and low precipitation, recharge rates decrease, the water level declines, and lakes dry up in springs. Over the last decades, the groundwater in the Nubian aquifer has dropped by 60 m [63,64]. Therefore, such depletion rate in Sahara is a call for the countries that share the Aquifer resources to develop long-term plans for sustainable management, to maximize their benefit.

The positive storage and high TWS annual amplitudes in West Africa, equatorial regions, and Zambezi regions are attributed to the path of the Intertropical Convergence Zone (ITCZ), which feeds these regions with significant precipitation during wet seasons. The ITCZ appears as thunderstorms that exhibit north-south shift. The ITCZ results from the convergence of trade winds in the northern and southern hemispheres and is governed by the Earth’s rotation and solar heating. In West Africa, the ITCZ causes rainy winds with unimodal and bimodal patterns. The unimodal rainy pattern is observed in Niger in August, whereas the bimodal pattern has been observed in one long rainy season in June followed by a short rainy season around October in Benin [65]. The dry season comes in winter (February) with the minimum amount of rain. In the Congo basin, annual precipitation results in two wet seasons and one dry season. The first rainy season begins in the north from January to May and moves to the South and the rest of the basin, followed by the dry season between May and September, and then back to the second rain season from September. In South Africa, the wet season is observed from December to April, and the dry season is between May and August [66]. TWS is recharged after the precipitation in the wet seasons in West Africa and Congo regimes. In the Volta basin, the precipitation is bound for TWS that changes at an annual scale with a phase lag of −0.42 ± 0.1 months [67]. The spatiotemporal variability of TWS and the declining or replenishing water level in lakes and water bodies have undergone climate change extremes (e.g., the Indian Ocean Dipole (IOD) and El Niño-Southern Oscillation) [68,69,70]. For example, half of the lost water in the Victoria Lake in last 80 years is attributed to climate conditions [71], and the 2006 to 2007 IOD fluctuations have replenished aquifer resources in Tanzania and East Africa [72].

## 5. Conclusions

In the African hydrological regimes, climate condition may contribute to the GRACE SH errors more than the size of regime does. Measurement and leakage errors increase with the aridity of the region’s area. The implemented scale factor based on the CLM4.0 model is also smaller for the arid regions than that for the humid regions. Hence, relying only on the scale factor in water storage estimation in arid zones from SH solutions is insufficient, especially when studying long-term groundwater depletion or water storage extremes. We find that using Slepian localization can efficiently reduce leakage errors because the optimal concentrated orthogonal basis cancels the leakage from outside the region. The method is highly suitable for estimating water storage in regions with low TWS content. TWS derived from the three estimates including SH, JPL-mascon, and Slepian localization solutions show better agreement in large-sized humid catchments in Africa. For medium and small-sized regions, Slepian, and JPL-mascon based estimate means are better predictors than the SH-based estimate. The long-term trend of TWS decreases in regions that are dominated by extensive irrigation, such as in the lower Nile and Sahara. Slepian estimation shows that TWS decreases by approximately −1.08 and −6.92 Gt/year in the lower Nile and Sahara, respectively. These rates are higher than those previously reported. Sahara loses TWS from transboundary aquifers between North African countries, thereby summoning the need for the long-term sustainable management of aquifer resources.

## Figures and Tables

**Table 1:** Characteristics of the 16 hydrological regimes under study in terms of area (A), aridity index (AI), as the ratio of the mean annual precipitation to the mean annual evapotranspiration. (CI) the climate classification of the regimes as AI ranges of <0.03, 0.03–0.2, 0.2–0.5, 0.5–0.65, and >0.65, for hyper-arid (HA), arid (A), semi-arid (SA), dry sub-humid (DSH), and humid (H), respectively. Leakage errors (σ*_l_*), measurement errors (σ*_m_*), scale factor for SH solutions (k_SH_), and gain factor for mascon solutions (k_MS_).

ROI ID	ROI Name	A (km^2^)	AI	Cl	σ*_l_* (mm)	σ*_m_* (mm)	Total Errors (mm)	*k*_SH_	*k*_MS_
**1**	Higher Nile	1,030,026	0.48	DSH	5.36	10.43	11.63	1.17	0.96
**2**	Lower Nile	455,637	0.15	A	13.25	25.43	28.62	0.38	0.88
**3**	Limpopo	415,017	0.33	SA	8.32	17.21	19.25	1.31	0.98
**4**	Middle Nile	1,901,388	0.28	SA	6.46	18.45	19.58	1.87	0.99
**5**	Niger	2,118,387	0.32	SA	5.74	12.34	13.54	1.16	1.59
**6**	Okavango	530,021	0.45	SA	6.85	16.86	18.15	1.12	1.23
**7**	Orange	973,218	0.31	SA	7.46	15.82	17.42	1.11	0.75
**8**	Sahara	6,907,746	0.02	HA	5.42	11.24	12.41	0.85	0.62
**9**	Volta	407,391	0.59	DSH	5.81	13.61	15.95	1.36	1.34
**10**	Zambezi	1,391,230	0.54	SH	7.41	16.42	17.93	1.11	1.01
**11**	Chad	2,662,435	0.19	A	4.92	10.82	11.82	0.99	1.05
**12**	Congo	4,014,741	0.89	H	5.38	10.83	12.36	1.24	1.09
**13**	Nile	3,241,937	0.29	SA	6.25	13.44	14.75	1.15	0.97
**14**	Rift valley	2,976,053	0.29	SA	5.34	9.53	10.91	0.96	0.72
**15**	South Africa	5,992,256	0.35	SA	4.55	12.82	13.53	0.49	0.28
**16**	West Africa	4,251,507	0.53	DSH	6.55	13.68	15.33	0.98	0.98

**Table 2:** Statistical intercomparison results of the three estimates in African hydrological regimes in terms of NSE and R. (NSE1, R1) for SL-TWS against SH-TWS, (NSE2 and R2) for SL-TWS versus MSC-TWS, and (NSE3 and R3) for SH-TWS against MSC-TWS.

ROI ID	ROI Name	NSE			R			No. of EOF	No. of Basis ≥ 0.6
		NSE1	NSE2	NSE3	R1	R2	R3		
**1**	Higher Nile	0.62	0.64	0.65	0.82	0.83	0.87	3	6
**2**	Lower Nile	0.53	−0.42	−1.02	0.75	0.25	0.38	2	2
**3**	Limpopo	0.46	0.03	0.08	0.73	0.52	0.52	2	2
**4**	Middle Nile	0.32	−0.32	−0.22	0.65	0.53	0.73	4	11
**5**	Niger	0.33	0.40	0.55	0.68	0.62	0.74	4	13
**6**	Okavango	0.42	0.21	−0.55	0.73	0.85	0.82	3	4
**7**	Orange	0.36	−0.07	−0.07	0.62	0.56	0.53	3	5
**8**	Sahara	0.52	0.31	0.09	0.77	0.74	0.64	2	35
**9**	Volta	0.67	0.52	0.64	0.85	0.78	0.75	3	2
**10**	Zambezi	0.73	−0.28	−0.10	0.89	0.73	0.78	3	8
**11**	Chad	0.57	−0.07	−0.07	0.82	0.85	0.74	3	14
**12**	Congo	0.60	0.50	0.30	0.88	0.87	0.73	4	29
**13**	Nile	0.80	0.70	0.40	0.92	0.96	0.88	3	19
**14**	Rift Valley	0.27	−3.09	−1.35	0.29	0.23	0.63	4	17
**15**	South Africa	0.82	0.60	0.70	0.95	0.92	0.93	3	35
**16**	West Africa	0.42	0.43	0.62	0.96	0.93	0.78	4	26

**Table 3:** Least square fitting results of the TWS time series for the three estimates in African hydrological regimes for long-term (trend), annual amplitude (AA), and semi-annual amplitude (SA).

ROI ID	ROI Name	SL-TWS			SH-TWS			MSC-TWS		
		Trend (cm/year)	AA (cm)	SA (cm)	Trend (cm/year)	AA (cm)	SA (cm)	Trend (cm/year)	AA (cm)	SA (cm)
**1**	Higher Nile	0.51	6.83	5.51	0.48	6.83	3.85	0.53	7.23	4.86
**2**	Lower Nile	−2.36	2.34	1.35	−2.10	2.14	0.57	−1.52	1.24	0.25
**3**	Limpopo	−0.54	3.82	1.65	−0.52	4.38	1.55	−0.43	4.65	1.76
**4**	Middle Nile	1.87	5.84	1.48	1.29	4.05	0.54	1.44	6.77	2.28
**5**	Niger	1.74	9.35	1.46	1.55	9.15	2.56	1.82	11.92	4.84
**6**	Okavango	1.44	3.72	1.84	1.63	3.67	0.64	1.50	6.89	0.45
**7**	Orange	0.35	1.65	0.52	0.31	1.65	1.25	0.34	1.64	2.11
**8**	Sahara	−1.02	0.44	0.14	−0.83	1.24	0.47	−1.31	0.09	0.32
**9**	Volta	2.82	14.30	3.45	4.75	13.9	1.69	4.83	13.21	0.27
**10**	Zambezi	1.35	14.91	3.27	1.45	9.98	3.28	1.64	14.34	3.58
**11**	Chad	0.05	8.53	0.65	0.06	6.52	1.84	0.07	8.79	1.49
**12**	Congo	0.05	5.64	2.18	0.05	5.13	1.55	0.01	5.76	3.57
**13**	Nile	0.13	3.25	0.27	0.26	2.65	0.17	0.28	3.92	1.17
**14**	Rift valley	0.06	1.64	1.46	0.07	1.54	2.35	0.08	1.53	1.66
**15**	South Africa	1.27	4.82	0.43	0.73	5.96	1.84	1.29	6.48	0.82
**16**	West Africa	1.71	9.89	0.45	1.63	12.5	0.81	1.73	13.24	3.95

## References

[1] Tapley B.D., Bettadpur S., Ries J.C., Thompson P.F., Watkins M.M. (2004). GRACE measurements of mass variability in the earth system. Science.

[2] Wahr J., Molenaar M., Bryan F. (1998). Time variability of the earth’s gravity field: Hydrological and oceanic effects and their possible detection using GRACE. J. Geophys. Res. Sol. Ea.

[3] Syed T.H., Famiglietti J.S., Rodell M., Chen J., Wilson C.R. (2008). Analysis of terrestrial water storage changes from GRACE and GLDAS. Water Resour. Res..

[4] Tapley B.D., Bettadpur S., Watkins M., Reigber C. (2004). The gravity recovery and climate experiment: Mission overview and early results. Geophys. Res. Lett..

[5] Ramillien G., Famiglietti J.S., Wahr J. (2008). Detection of continental hydrology and glaciology signals from GRACE: A review. Surv. Geophys..

[6] Schmidt R., Schwintzer P., Flechtner F., Reigber C., Guntner A., Doll P., Ramillien G., Cazenave A., Petrovic S., Jochmann H. (2006). GRACE observations of changes in continental water storage. Glob. Planet. Chang..

[7] Rodell M., Chen J.L., Kato H., Famiglietti J.S., Nigro J., Wilson C.R. (2007). Estimating groundwater storage changes in the Mississippi River Basin (USA) using GRACE. Hydrogeol. J..

[8] Yeh P.J.F., Swenson S.C., Famiglietti J.S., Rodell M. (2006). Remote sensing of groundwater storage changes in Illinois using the gravity recovery and climate experiment (GRACE). Water Resour. Res..

[9] Voss K.A., Famiglietti J.S., Lo M., Linage C., Rodell M., Swenson S.C. (2013). Groundwater depletion in the Middle East from GRACE with implications for transboundary water management in the Tigris-Euphrates-Western Iran region. Water Resour. Res..

[10] Rodell M., Velicogna I., Famiglietti J.S. (2009). Satellite-based estimates of groundwater depletion in India. Nature.

[11] Rodell M., Famiglietti J.S., Chen J., Seneviratne S.I., Viterbo P., Holl S., Wilson C.R. (2004). Basin scale estimates of evapotranspiration using GRACE and other observations. Geophys. Res. Lett..

[12] Ramillien G., Frappart F., Guntner A., Ngo-Duc T., Cazenave A., Laval K. (2006). Time variations of the regional evapotranspiration rate from gravity recovery and climate experiment (GRACE) satellite gravimetry. Water Resour. Res..

[13] Chen J.L., Wilson C.R., Tapley B.D. (2010). The 2009 exceptional amazon flood and interannual terrestrial water storage change observed by GRACE. Water Resour. Res..

[14] Thomas A.C., Reager J.T., Famiglietti J.S., Rodell M. (2014). A GRACE-based water storage deficit approach for hydrological drought characterization. Geophys. Res. Lett..

[15] Reager J.T., Thomas B.F., Famiglietti J.S. (2014). River basin flood potential inferred using GRACE gravity observations at several months lead time. Nat. Geosci..

[16] Syed T.H., Famiglietti J.S., Chambers D.P. (2009). GRACE-based estimates of terrestrial freshwater discharge from basin to continental scales. J. Hydrometeorol..

[17] Syed T., Famiglietti J., Chen J., Rodell M., Seneviratne S., Viterbo P., Wilson C. (2005). Total basin discharge for the Amazon and Mississippi River basins from GRACE and a land-atmosphere water balance. Geophys. Res. Lett..

[18] Tang J.S., Cheng H.W., Liu L. (2014). Assessing the recent droughts in southwestern China using satellite gravimetry. Water Resour. Res..

[19] Liu R., Li J., Fok H.S., Shum C.K., Li Z. (2014). Earth surface deformation in the north China plain detected by joint analysis of GRACE and GPS data. Sensors.

[20] Zou R., Wang Q., Freymueller J.T., Poutanen M., Cao X., Zhang C., Yang S., He P. (2015). Seasonal hydrological loading in southern Tibet detected by joint analysis of GPS and GRACE. Sensors.

[21] Van Dam T., Wahr J., Lavallée D. (2007). A comparison of annual vertical crustal displacements from GPS and gravity recovery and climate experiment (GRACE) over Europe. J. Geophys. Res. Sol. Ea.

[22] Lo M.H., Famiglietti J.S., Yeh P.J.F., Syed T.H. (2010). Improving parameter estimation and water table depth simulation in a land surface model using GRACE water storage and estimated base flow data. Water Resour. Res..

[23] Guntner A. (2008). Improvement of global hydrological models using GRACE data. Surv. Geophys..

[24] Tapley B.D., Schutz B.E., Born G.H. (2004). Statistical Orbit Determination.

[25] Watkins M.M., Wiese D.N., Yuan D.N., Boening C., Landerer F.W. (2015). Improved methods for observing earth’s time variable mass distribution with GRACE using spherical cap mascons. J. Geophys. Res. Sol. Ea.

[26] Rowlands D.D., Luthcke S.B., McCarthy J.J., Klosko S.M., Chinn D.S., Lemoine F.G., Boy J.P., Sabaka T.J. (2010). Global mass flux solutions from GRACE: A comparison of parameter estimation strategies-mass concentrations versus stokes coefficients. J. Geophys. Res. Sol. Ea.

[27] Wahr J., Swenson S., Velicogna I. (2006). Accuracy of GRACE mass estimates. Geophys. Res. Lett..

[28] Longuevergne L., Scanlon B.R., Wilson C.R. (2010). GRACE hydrological estimates for small basins: Evaluating processing approaches on the high plains aquifer, USA. Water Resour. Res..

[29] Swenson S., Wahr J. (2006). Post-processing removal of correlated errors in GRACE data. Geophys. Res. Lett..

[30] Jekeli C. (1981). Alternative Methods to Smooth the Earth's Gravity Field.

[31] Werth S., Guntner A., Schmidt R., Kusche J. (2009). Evaluation of GRACE filter tools from a hydrological perspective. Geophys. J. Int..

[32] Swenson S.C., Wahr J.M. (2011). Estimating signal loss in regularized GRACE gravity field solutions. Geophys. J. Int..

[33] Klees R., Zapreeva E., Winsemius H., Savenije H. (2006). The bias in GRACE estimates of continental water storage variations. Hydrol. Earth Syst. Sci..

[34] Landerer F.W., Swenson S.C. (2012). Accuracy of scaled GRACE terrestrial water storage estimates. Water Resour. Res..

[35] Harig C., Simons F.J. (2012). Mapping greenland’s mass loss in space and time. Proc. Natl. Acad. Sci. USA.

[36] Simons F.J., Dahlen F.A. (2006). Spherical slepian functions and the polar gap in geodesy. Geophys. J. Int..

[37] Wang L., Shum C.K., Simons F.J., Tapley B., Dai C.L. (2012). Coseismic and postseismic deformation of the 2011 Tohoku-Oki earthquake constrained by GRACE gravimetry. Geophys. Res. Lett..

[38] Han S.C., Riva R., Sauber J., Okal E. (2013). Source parameter inversion for recent great earthquakes from a decade-long observation of global gravity fields. J. Geophys. Res. Sol. Ea.

[39] Wiese D.N., Nerem R.S., Han S.C. (2011). Expected improvements in determining continental hydrology, ice mass variations, ocean bottom pressure signals, and earthquakes using two pairs of dedicated satellites for temporal gravity recovery. J. Geophys. Res. Sol. Ea.

[40] Plattner A., Simons F.J. (2015). High-resolution local magnetic field models for the Martian South Pole from Mars Global Surveyor Data. J. Geophys. Res. Planets.

[41] Vorosmarty C.J., Green P., Salisbury J., Lammers R.B. (2000). Global water resources: Vulnerability from climate change and population growth. Science.

[42] World Health Organization (2014). Progress on Sanitation and Drinking Water: 2014 Update.

[43] FAO World Map of the Major Hydrological Basins. http://www.fao.org/geonetwork/.

[44] Trabucco A., Zomer R. Global Aridity Index (Global-Aridity) and Global Potential Evapo-Transpiration (Global-Pet) Geospatial Database. http://www.cgiar-csi.org/.

[45] Trabucco A., Zomer R.J., Bossio D.A., van Straaten O., Verchot L.V. (2008). Climate change mitigation through afforestation/reforestation: A global analysis of hydrologic impacts with four case studies. Agric Ecosyst. Environ..

[46] GRACE Tellus. http://GRACE.Jpl.Nasa.Gov/mission/GRACE/.

[47] Simons F.J., Dahlen F.A., Wieczorek M.A. (2006). Spatiospectral concentration on a sphere. SIAM Rev..

[48] Simons F.J., Hawthorne J.C., Beggan C.D. (2009). Efficient analysis and representation of geophysical processes using localized spherical basis functions. Proc. SPIE.

[49] Simons F., Freeden W., Nashed M.Z., Sonar T. (2010). Slepian functions and their use in signal estimation and spectral analysis. Handbook of Geomathematics.

[50] Cheng M.K., Ries J.C., Tapley B.D. (2011). Variations of the earth’s figure axis from satellite laser ranging and GRACE. J. Geophys. Res. Sol. Ea.

[51] Swenson S., Chambers D., Wahr J. (2008). Estimating geocenter variations from a combination of GRACE and ocean model output. J. Geophys. Res. Sol. Ea.

[52] Geruo A., Wahr J., Zhong S.J. (2013). Computations of the viscoelastic response of a 3-d compressible earth to surface loading: An application to glacial isostatic adjustment in Antarctica and Canada. Geophys. J. Int..

[53] Jolliffe I. (2002). Principal Component Analysis.

[54] Nash J.E., Sutcliffe J.V. (1970). River flow forecasting through conceptual models part I—A discussion of principles. J. Hydrol..

[55] Okavango Basin Information System (OBIS). http://leutra.Geogr.Uni-jena.De/obis/metadata/start.Php.

[56] Long D., Longuevergne L., Scanlon B.R. (2015). Global analysis of approaches for deriving total water storage changes from GRACE satellites. Water Resour. Res..

[57] Chen J.L., Wilson C.R., Famiglietti J.S., Rodell M. (2007). Attenuation effect on seasonal basin-scale water storage changes from GRACE time-variable gravity. J. Geod..

[58] Dutt Vishwakarma B., Devaraju B., Sneeuw N. (2016). Minimizing the effects of filtering on catchment scale GRACE solutions. Water Resour. Res..

[59] Wiese D.N., Landerer F.W., Watkins M.M. (2016). Quantifying and reducing leakage errors in the JPL RL05M GRACE mascon solution. Water Resour. Res..

[60] Sultan M., Ahmed M., Wahr J., Yan E., Emil M. (2014). Monitoring aquifer depletion from space: Case studies from the saharan and arabian aquifers. Remote Sensing of the Terrestrial Water Cycle.

[61] Ramillien G., Frappart F., Seoane L. (2014). Application of the regional water mass variations from GRACE satellite gravimetry to large-scale water management in Africa. Remote Sens..

[62] Goncalves J., Petersen J., Deschamps P., Hamelin B., Baba-Sy O. (2013). Quantifying the modern recharge of the “fossil” Sahara aquifers. Geophys. Res. Lett..

[63] Foster S., Loucks D.P. (2006). Non-Renewable Groundwater Resources: A Guidebook on Socially-Sustainable Management for Water-Policy Makers.

[64] Gleeson T., VanderSteen J., Sophocleous M.A., Taniguchi M., Alley W.M., Allen D.M., Zhou Y.X. (2010). Groundwater sustainability strategies. Nat. Geosci..

[65] Sultan B., Janicot S. (2000). Abrupt shift of the ITCZ over West Africa and intra-seasonal variability. Geophys. Res. Lett..

[66] Asnani G. (1993). Tropical Mmeteorology.

[67] Ferreira V.G., Andam-Akorful S.A., He X.-F., Xiao R.-Y. (2014). Estimating water storage changes and sink terms in volta basin from satellite missions. Water Sci. Eng..

[68] Awange J.L., Forootan E., Kusche J., Kiema J.B.K., Omondi P.A., Heck B., Fleming K., Ohanya S.O., Goncalves R.M. (2013). Understanding the decline of water storage across the Ramser-Lake Naivasha using satellite-based methods. Adv. Water Resour..

[69] Becker M., LLovel W., Cazenave A., Guntner A., Cretaux J.F. (2010). Recent hydrological behavior of the East African great lakes region inferred from GRACE, satellite altimetry and rainfall observations. C. R. Geosci..

[70] Gan T., Ito M., Huelsmann S., Qin X., Lu X., Liong S., Rutschman P., Disse M., Koivosalo H. (2015). Possible climate change/variability and human impacts, vulnerability of african drought prone regions, its water resources and capacity building. Hydrol. Sci. J..

[71] Swenson S., Wahr J. (2009). Monitoring the water balance of Lake Victoria, East Africa, from space. J. Hydrol..

[72] Deus D., Gloaguen R., Krause P. (2013). Water balance modeling in a semi-arid environment with limited in situ data using remote sensing in Lake Manyara, East African Rift, Tanzania. Remote Sens..

[73] Harig C., Lewis K.W., Plattner A., Simons F.J. (2015). A suite of software analyzes data on the sphere. Eos.

